# Factors influencing student’s specialty choices in Lomé faculty of medicine (Togo)

**DOI:** 10.1186/s12909-021-03063-2

**Published:** 2021-12-14

**Authors:** Julienne Noude Teclessou, Aminou Dabouda, Sefako Akakpo, Panawe Kassang, Bayaki Saka, Koussake Kombate, Palokinam Pitche

**Affiliations:** Department of Dermatology, University Teaching Hospital of Lomé. Faculty of health sciences, university of Lome (Togo), Lomé, Togo

**Keywords:** Specialties, Medical students, Factors influencing

## Abstract

**Background:**

The choice of specialty in medicine is an important decision for the individual, but also for health system. This choice combined personals reasons, professional desires and needs of the health system. The number of specialists in the country depends of this choice. Very few studies have focused on factors influencing the choice of specialties among medical students in Africa. Also, in the absence of specialist needs planning in Togo. This study, aims to determine the factors influencing the choice of specialty among students at the Faculty of Health Sciences of the University of Lomé (FSS-UL).

**Methods:**

This was a descriptive cross-sectional study that took place from June 1 to June 30, 2019 with medical students of the doctoral cycle and doctors in specialization studies in the various Diploma of Special Study (DSS) available at the FSS-UL.

Data collection was done at the surveyed’s training sites. Pre-established and pre-tested fact sheets were giving and explained to the students by data collection team. Data collection team return at the surveyed’s training sites 72 h after to collect pre-established fact sheets. Following variables study including: factors (individual; related to the medical curriculum); the advantages and attractiveness of the specialty that can influence students’ choice. Data analysis was carried out using Epi Info 6.0 software. The significance threshold was 5%.

**Results:**

At the time of the survey, the FSS-UL had 147 doctoral students and 211 specialty students. A total of 251 participants responded to the questionnaires. These included 140 doctoral students and 111 specialty students. The choice of specialties requiring night work such as gynecology, surgery was significantly associated with the male sex (*p* = 0.001). There was significant association between having financial support (*p* = 0.001), remuneration related to the specialty (*p* = 0.0001) and the decision to beginning specialty studies immediately completing general medical studies. Interest in lectures (*p* = 0.003), teacher support as a mentor in the specialty (*p* = 0.01) and easy accessibility to teachers (*p* = 0.008) were medical curriculum factors significantly associated with specialty choice. Facility to work in public and private sector was mentioned by 55.3% of respondents who chose gynecology (*p* = 0.03).

Interest in lectures (*p* = 0.003), was significantly associated with choice of fundamental sciences; and work in international fields was significantly associated with the choice of pediatric and public health (*p* = 0.0001).

**Conclusion:**

Factors influencing the choice of certain specialty were balance between family and professional life; financial support to studies, the remuneration opportunities related to the specialty, access to university career. Intervention on these factors will allow a balance between the numbers of doctors trained in the different specialties.

## Introduction

The Diploma of Special Study (DSS) is a post-doctoral medical training allowing general practitioners who desire to practice in the specialty of their choice. This is training that enables each country to meet its needs in terms of health personnel. The choice of specialty in medicine is an important decision for the individual, but also for the future workforce in the health system, especially in underdeveloped countries. In some countries, such as France, medical students’ access to different specialties is conditioned by national needs. Medical students wishing to practice a specialty therefore pass a competition (National qualifying Exam) at the beginning of the doctoral cycle [1]. The number of places available in each specialty is defined in advance by national needs. Students who have passed this competition choose their specialty in order of merit. This may require some students to make a specialty that is not their choice given the rank occupied in this competition.

In Togo, there is no planning policy regarding the need for health personnel. Doctors are therefore free to register in a specialty of their choice whenever they want. When the desired specialty does not exist in the country, doctors often go to another country in the sub-region to be trained in that desired specialty. This phenomenon can lead to a high number of specialists in some specialties at the expense of others.

At the Faculty of Health Sciences of the University of Lomé (FSS-UL), the entry into specialization for DSS is not conditioned by a competition but by the holding of a doctoral degree in medicine. The registration fee is about 800 euros for Togolese and 1500 euros for students of other nationalities [2]. The training lasts 4 years to 5 years is unpaid, according to specialties. Medical specialists may work in private or integrated public service if national services wish to integrate them.

The first DSS in Togo (pediatrics, general surgery) was created in 1988, at the FSS-UL [3–5]. At the start of the 2017–2018 academic years, the FSS-UL had 13 DSS including the medical specialty; surgery and surgical specialties; pediatrics and gynecology. Despite this increase in specialty at FSS-UL, some DSS still non-existent in Togo (neurosurgery, oncology, vascular surgery).

Although some students already have a preference for certain specialties prior to entering the FSS, most do not choose their specialty until the end of their general practice course or in the early years of their position. Several factors can influence this choice: individual characteristics, perceived benefits and the attractiveness of certain specialties, factors associated with medical curricula [6–8].

Given the lack of health needs planning on the one hand, and the lack of financial support for specialty physicians during their training, the choice of specialty could be strongly influenced by individual factors. Also, in the absence of specialist needs planning in Togo, knowledge of the factors influencing the choice of specialty among physicians will help to determine why physicians choose specialties at the expense of others; and take steps to ensure good coverage of the various specialists in health regions.

This study, the first in Togo, aims to study the factors influencing the choice of specialty among students at FSS-UL in Togo.

## Method

### Study design/setting

This was a descriptive cross-sectional study that took place from 1 to 30 June 2019 with medical students of the doctoral cycle (DS3-DS4) and doctors in specialization studies at the FSS-UL.

### Study participants and sampling

The study concerned the medical students of the doctoral cycle (DS3-DS4) and doctors in specialization studies in the various DSS available at the FSS-UL.

In Togo, access to medical studies is done after validation of secondary education. Medical studies are divided into two large parts based on the French model:

- The first part: general medicine that lasts 7 school years (each year divided into 2 semesters) and is divided into three cycles: the license (which lasts 3 years: LS1-LS2; LS3-LS4 and LS5-LS6); the master’s degree (which lasts 2 years: MS1-MS2; MS3-MS4) and the doctoral cycle (which lasts 2 years: DS1-DS2; DS3-DS4). His various cycles are sanctioned by only one degree: the doctor of medicine diploma obtained at the end of the doctoral cycle. The regulators of this diploma can therefore freely practice general medicine, family medicine.
- The second part: specialty studies. It’s optional. Access to this training is conditioned by obtaining a medical doctor’s degree.

It was noted that training in both general medicine and specialty is entirely the responsibility of students (or their parents) and that students receive no remuneration during the various courses.

The sampling was exhaustive. All phD students (DS3-DS4) and all doctors in specialization studies in the various DSS, present in the city of Lomé at the time of data collection were included. We did not include doctoral students (DS3-DS4) or physicians specializing in internships in another city in the country or outside the country at the time of the survey. Also, general practitioners present in specialty services but not enrolled in DSS for the academic year of data collection were not included.

### Data collection

Data collection was done at the surveyed’s training sites. Data were collected using a standardized questionnaire in French.

We have set up a data collection team composed by medical students in the master’s cycle. The questionnaire was explained to the data collection team. Before beginning survey, questionnaire was pretested with two medical students of doctoral cycle.

The questionnaire including:

- Sociodemographic: age, sex, nationality, function, marital status, occupation of parents.
- Medical and specialty studies: funding for studies; specialty envisages at the beginning of medical studies and current specialty.
- Individual factors influencing the choice: the delay between the end of general medicine studies and the beginning of the specialty; work-life balance Skills and skills of their own potential income related to the specialty.
- Factors related to the medical curriculum: previous internship in the chosen specialty service; influence of teachers.
- Factors related to the advantages and attractiveness of the specialty: teamwork; Potential specialty-related income career prospects.

### Analysis

The data analysis was done with the Epi Info 6.0 software. The uni-varied analysis was done between the different specialties and the factors influencing their choices. The threshold for significance was 5%.

### Ethical considerations

The entire study protocol (including questionnaires and consent forms; Ref No: 0114/2019/MS/CAB/DGS/DPLET/CBRS) were approved by the board committee of the Bioethics Committee of the Ministry of Health.

We obtained consent from study subjects that participated in the study. For each of the FSWs surveyed, the objectives, benefits to participate in the survey were clearly stated them.

The anonymity of the participants was preserved. The informing consent of the participants was obtained. The participation of the respondents was free and informed.

## Results

At the time of the survey, the FSS-UL had 147 doctoral students and 211 specialty students. A total of 251 of the 358 doctoral students/specialty physicians responded to the questionnaires, representing a response rate of 70.1%. The response rate for doctoral students was 95.2% (140/147) and 52.6% for DSS students (111/ 211).

The average age of the participants was 29.3 years and − 12.5 (extremes from 22 to 49 years of age). The sex ratio (M/F) was 3.4. The majority of participants 184 (73.9%) were of Togolese nationalities; follow-up of Nigerian nationalities 30 (12.0%) Cameroonian 15 (6.0%). General medicine studies were funded for the majority of respondents (185/251 or 73.7%) by the parents. Of the 251 surveyed, 2 (0.8%) did not consider specializing. 103/249 (41.4%) had started or would like to begin specialization studies in the school year following the completion of general medicine studies. Self-financing was the primary source of funding for specialty studies by 66.2% of doctoral students and 52.3% of physicians in DSS respectively. Sixty three (56.7%) DSS students chose a different specialty from the one envisaged at the beginning of their medical study. Also, 98 (70.0%) phD students are considering changing their initial choice.

Medical specialties were the most chosen by respondents (98 /249; 39.3%), followed by surgical specialties (65/249; 26.1%) and gynecology (47/249; 18.9%) (Table 1). The most chosen medical specialties were cardiology (32/98 or 12.8%) follow-up of radiology and medical imaging (17/98 or 6.8%). It is a medical specialty available in Togo (Table 1) Ophthalmology (19/65 or 7.3%) traumatology (16/65 or 6.4%) were the first-class surgical specialties.

**Table 1:** Existing specialty in Togo and the choice of participants

Specialty chosen by respondents	N (%)	Existence of the specialty in Togo
Medical specialty	**98 (39,36)**	
Cardiology	32 (12,85)	Yes
Radiology and medical imaging	17 (6,83)	Yes
Internal medicine	10 (4,02)	Yes
Dermatology	9 (3,61)	Yes
Pneumology-phtisiology	8 (3,21)	Yes
Neurology	6 (2,41)	Yes
Rheumatology	4 (1,61)	No
Endocrinology	3 (1,20)	No
Legal medicine	2 (0,81)	No
Nephrology	2 (0,81)	No
Anesthesia- and intensive care	1 (0, 40)	No
Geriatrics	1 (0, 40)	No
Hepato-Gastro-Enterology	1 (0, 40)	No
Infectious disease	1 (0,40)	No
Occupational medicine	1 (0, 40)	No
**Surgical specialties**	**65 (26,1)**	
Ophthalmology	19 (7,63)	Yes
Traumatology	16 (6,42)	Yes
General surgery	13 (5,22)	Yes
Pediatric surgery	8 (3,21)	Yes
Otorhinolaryngology	7 (2,81)	Yes
Visceral surgery	1 (0, 40)	No
Neurosurgery	1 (0, 40)	No
**Gynecology**	**47 (18,88)**	Yes
**Fundamental sciences**	**15 (6,02)**	
Biology	10 (4,02)	No
Hematology	5 (2)	No
**Public health**	**14 (5,62)**	Yes
**Pediatric**	**10 (4,02)**	Yes

### Individual factors influencing the choice of specialty

Specialty physicians begin their specialty study on average 2.8/− 1.6 years (extreme 1 to 8 years) after completing general medical studies, while doctoral students were considering starting specialty studies on average 3.0/− 1.7 years (extreme 1 to 10 years) after graduation.

A few reasons cited by respondents beginning or wanting to begin their specialization studies at the end of general medicine studies were quickly completing their specialty studies (*p* = 0.001), being paid as a specialist (*p* = 0.0001) (Table 2).

**Table 2:** Factors influencing the beginning of post graduate cycle

	Immediately* (*N* = 103)	lately** (*N* = 146)	both*** (*N* = 249)	*P*-Value
	n (%)	n (%)	n (%)	
Have a financial support	74 (71.8)	73 (50.0)	147 (59.0)	0.001
No financial support	29 (28.2)	73 (50.0)	102 (41.0)	
Practice as a general practitioner	6 (5.8)	78 (53.4)	84 (33.7)	0.0001
Practice as a specialist	40 (38.8)	6 (4.1)	46 (18.5)	
Rapidly complete post graduate cycle	69 (67.0)	14 (9.6)	83 (33.3)	0.0001
Have the salary of a specialist	41 (39.8)	17 (11.6)	58 (23.3)	0.0001

* Respondents that start specialty studies academic year following ending their studies in general practitioner ** Respondents who began specialty studies one or more years after completing general medical studies *** all respondents

The absence of a life-threatening emergency (*p* = 0.0001) of advantageous exercise hours (*p* = 0.003) was associated with the choices of basic sciences. Contact with patients had motivated the choice of surgical specialties and gynecology among respondents (*p* = 0.0001) (Table 3).

**Table 3:** Individuals factors influencing the choice of a specialty

	Medical specialties *N* = 98 n (%)	Surgical specialties *N* = 65 n (%)	gynecology *N* = 47 n (%)	Fundamental sciences *N* = 15 n (%)	Public health *N* = 14 n (%)	Pediatric *N* = 10 n(%)	*P*-value
**Individuals factors: balance between work and personal life**							
No emergencies	26 (24.1)	15 (23.1)	0 (0)	8 (53.3)	7 (50)	0 (0)	0.0001
Absence of custody	27 (25)	16 (24.6)	0 (0)	8 (53.3)	7 (50)	0 (0)	0.0001
Advantageous hours of work	30 (27.8)	18 (27.7)	3 (6.4)	7 (46.7)	5 (35.7)	0 (0)	0.003
Capable of night work	21 (20.4)	23 (35.4)	21 (44.7)	0 (0)	1 (7.1)	1 (10.00)	0.001
Spouse’s advice	6 (5.6)	3 (4.6)	6 (12.8)	1 (6.7)	0 (0)	0 (0)	0.3
Family constraint	12 (11.1)	7 (10.8)	7 (14.9)	0 (0)	0 (0)	0 (0)	0.3
**Individual factors**: **personal skills and competences**							
Personal skills	71 (65.7)	44 (67.7)	35 (74.5)	14 (93.3)	11 (78.6)	9 (90.0)	0.2
Known as easy specialty	2 (1.9)	2 (3.1)	3 (6.4)	0 (0)	1 (7.1)	0 (0)	0.5
Physical inability to exercise another specialty	4 (3.7)	1 (1.5)	0 (0)	0 (0)	1 (7.1)	0 (0)	0.4
Personal inability to exercise another specialty	2 (1.9)	2 (3.1)	1 (2.1)	1 (6.7)	0 (0)	0 (0)	0.7
**Individuals factors: Contact with patients**							
Be in contact with patients	83 (76.9)	60 (92.3)	41 (87.2)	3 (20)	2 (14.3)	10 (100)	0.0001
Avoid contact with patients	10 (9.3)	2 (3.1)	0 (0)	5 (33,3)	7 (50)	0 (0)	0.0001
**Personal financial factors**							
Default choice	15 (13.9)	4 (6.2)	7 (14.9)	1 (6.7)	1 (7.1)	0 (0)	0.4
Lack of financial support	10 (9.3)	12 (18.5)	5 (10,6)	2 (13.3)	1 (7.1)	0 (0)	0.4
Post graduate cycle not available in my country	10 (9.3)	2 (3.1)	0 (0)	3 (20.0)	2 (14.3)	0 (0)	0.02

The unavailability of the specialties of choice in the country had motivated 20.0% of respondents to choose the specialties of basic sciences (*p* = 0.02) because the specialty of their choice was not available in the country.

### Factors related to medical curriculum

Interest in lectures (*p* = 0.003), teacher support as a mentor in the specialty (*p* = 0.01), easy accessibility to teachers (*p* = 0.008) were factors related to the medical curriculum significantly associated with specialty choices (Table 4).

**Table 4:** Professional factors influencing the choice of specialty

	Medical specialties *N* = 98 n (%)	Surgical specialties *N* = 65 n (%)	gynecology *N* = 47 n (%)	Fundamental sciences *N* = 15 n (%)	Public heath *N* = 14 n (%)	Pediatric *N* = 10 n(%)	*P*-value
**Factors related to medical training**							
Anterior training in the team	33 (30,6)	13 (20)	2 (4.3)	6 (40)	11 (78.6)	10 (100)	0.1
On a professor advice	15 (13.9)	8 (12.3)	6 (12.8)	1 (6.7)	3 (21.4)	0 (0)	0.8
Mentor in the specialty	8 (7.4)	13 (20)	7 (14.9)	6 (40.0)	1 (7.1)	1 (10.0)	0.01
Impress by a Teacher	30 (27.8)	18 (27.7)	20 (42.6)	8 (53.3)	6 (42.9)		0.1
Interest by the lecture	24 (22.2)	27 (41.5)	12 (25.5)	10 (66.7)	4 (28.6)	1 (10.0)	0.003
Easy access to teachers	41 (38.0)	19 (29.2)	5 (10.6)		7 (50)	2 (20.0)	0.008
**Factors related to advantages of specialty**							
**Team work and renown of the specialty**							
Work in team	33 (30.6)	31 (47.7)	19 (40.4)	1 (6.7)	8 (57.1)	1 (10.0)	0.008
Prestigious specialty	31 (28.7)	17 (26.2)	10 (21.3)	3 (20)	4 (28.6)	1 (10.0)	0.8
Binding specialty	8 (7.4)	5 (7.7)	8 (17)	0 (0)	0 (0)	0 (0)	0.1
**Potential income**							
Good salary	12 (11.1)	14 (21.5)	11 (23.4)	5 (33.3)	6 (42.9)	1 (10.0)	0.01
Work in private sector	48 (44.4)	37 (56.9)	31 (66)	7 (46.7)	3 (21.4)	1 (10.0)	0.01
Work in public and private sector	43 (39.8)	26 (40)	26 (55.3)	6 (40)	1 (7.1)	3 (30.0)	0.03
**Access to academic career**							
Access to public service	19 (17.6)	17 (26.2)	11 (23.4)	7 (46.7)	4 (28.6)	1 (10.0)	0.1
Work in international fields	33 (30.6)	26 (40)	12 (25.5)	10 (66.7)	11 (78.6)	8 (80.0)	0.0001
Access to academic career	21 (19.4)	19 (29.2)	3 (6.4)	7 (46.7)	2 (14.3)	0 (0)	0.003
Lack of specialist in this field	21 (19.4)	16 (24.6)	0 (0)	6 (40)	2 (14.3)	0 (0)	0.002

### Factors related to the benefits and attractiveness of the specialty

Teamwork (*p* = 0.008) and attractive compensation opportunities (*p* = 0.01) were significantly associated with the choice of specialties, particularly public health (Table 3). The choice of basic science specialties was associated with the envy of access to a university career (*p* = 0.003) (Table 4).

## Discussion

The results of this study show that the absence of certain specialties in the country and the lack of financial support forces general practitioners to choose specialties that are only available in Togo (gynecology, pediatrics, general surgery, medical specialty and others).

This leads progressively to a high number of certain specialists and the almost total absence of other specialists in the national health system. Other individual factors that influence the choice of specialties are: work-life balance, interest in lectures and the accessibility of teachers, compensation opportunities and access to university careers.

We noted a low participation rate among DSS (52.6%). Gaucher [9] in a study near medical students in France, had noted a participation rate of 24%. The average age of participants was 29.3 years. This is similar to those found by other authors (21.1 to 24 years) [10, 11]. We noted a male predominance in contrast to France or Morocco where a female predominance was noted: (57 to 67%) [9–12].

The lack of financial means and the non-availability of certain specialties in the country significantly influence the choice of specialty by medical students in Togo.

In fact, only 0.8% of respondents decided not to study a specialty later. There is therefore a willingness among general practitioners to study a specialty. However, an average age of 3 years (with extremes of up to 10 years) is noted between the end of general practitioner studies and the beginning of the specialty. This long period may be explained by the lack of means to self-finance one’s specialty studies just after finishing general practitioner studies. Having financial support was significantly associated with the decision to start a specialty right after completing general medical school.

The decision to begin his specialty studies the year after the end of medical studies was influenced by the desire to quickly finish specialty studies (*p* = 0.0001); follow-up on remuneration as a specialist (*p* = 0.0001). Compensation is therefore an important part of career decision-making. In a study conducted by Ekouevi in sub-Saharan French-speaking African countries, 39.9% of students who wanted to specialize in public health had mentioned salary incentives and other social benefits [13]. Also, the long enough duration of medical studies/specialties justifies this choice to quickly finish.

We noted a clear predominance of choice of medical specialties (39.0%), followed by surgical specialties (25.9%). Boutgayout [11] in Morocco, Cleland [12] in Scotland had also found a predominance of medical specialties (61 and 59.2%) respectively. But the predominant choice of certain specialties (gynecology-obstetrics, cardiology, ophthalmology) at the expense of others in our call on the health authorities to take measures that can facilitate doctors’ access to other specialties. The predominant choice of these specialties can be explained by their availability in the country.

In the absence of any funding for general medicine studies and specialty studies in our country, enrolling doctors in specialties that require them to leave the country is not always easy. Doctors can therefore enroll in specialties by default, which may limit the national coverage of different specialties. The lack of funding during medical studies in Togo also justifies the opportunities for significant remuneration in certain specialties mentioned by the respondents. Choucair in Lebanon [14] had not found a link between the choice of specialty and income.

56.7% of doctors in DSS do not currently study the specialty they were considering at the beginning of their medical study. Our results are close to those of Gaucher [9] in France had reported a change in the choice of specialties in 60% of students up to three times between the beginning of their medical course and the sixth year

Other factors influencing the choice of specialties among medical students in Togo seem to be common to other countries. These are the absence of a vital emergency in the specialty and the desire to access the university career.

The absence of a vital emergency and care (*p* = 0.0001) in the specialty and advantageous hours of exercise (*p* = 0.003) was significantly associated with the selection of basic science specialties. Gaucher *in* France [9] had found similar results or 56% of participants had chosen the specialties allowing them to have a quality of professional and personal life. Also, 73% of interns in Morocco chose specialties that allowed them to balance personal and professional. The choice of basic science specialties (93.3%), public health (78.5%) or gynecology (74.5%) was guided by the personal skills of the respondents. Our results are superior to those of Kadher [15] who found that 64% of study participants believe that their own skills and skills allow them to choose their specialty.

Contact with patients was significantly associated with the choice of surgery (92.3%) and gynecology (87.2%) (*p* = 0.0001). In Morocco [11], 17% of interns chose specialties such as biology, radiology to avoid contact with patients or with medical staff.

There was a significant association between the choice of basic science specialties and the envy of an academic career (*p* = 0.003). Indeed, the scarcity of teachers in certain disciplines of basic sciences such as medical biochemistry, parasitology in Togo may lead some students to think of entering the university career by doing these specialties. In France, 17% of students chose their specialties for academic interest [9]. Boutgayout in Morocco [11] had found that the desire for university and continuing education was associated with medical specialties.

### Limitations

The main limitation of this study is that some participants had already begun their specialty studies but others were in the doctoral cycle. This makes it impossible to have the same views of the two groups of participants on the issue.

Furthermore, the fact that this study is a cross-sectional study is also a limitation. The number medical students of the doctoral cycle (DS3-DS4) and doctors in specialization studies change over year. This study is an opinion survey. So, the variation in the opinions of the respondents can change the conclusions of this study.

## Conclusion

Several individual factors influence the choice of certain specialties at the expense of others by doctors in Togo. It is essentially about the balance between family and professional life, the availability of the specialty in the country, the remuneration opportunities related to the specialty, access to university career. In the absence of a training policy, we suggest intervention on these factors will allow a balance between the numbers of doctors trained in the different specialties.

## Data Availability

The datasets used and/or analyzed during the current study available from the corresponding author on reasonable request.

## Abbreviations

DSS: Diploma of Special Study
FSS-UL: Faculty of Health Sciences of the University of Lomé
